# Preparing intensive care for the next pandemic influenza

**DOI:** 10.1186/s13054-019-2616-1

**Published:** 2019-10-30

**Authors:** Taylor Kain, Robert Fowler

**Affiliations:** 1 Department of Critical Care, University of Toronto, Toronto, ON Canada; 2 Sunnybrook Health Sciences Centre, Room D478, 2075 Bayview Avenue, Toronto, ON M4N 3M5 Canada

**Keywords:** Influenza, Pandemic, Intensive care, Preparation, Resource allocation, Highly pathogenic avian influenza, Human, Health care worker safety, Triage, Research

## Abstract

Few viruses have shaped the course of human history more than influenza viruses. A century since the 1918–1919 Spanish influenza pandemic—the largest and deadliest influenza pandemic in recorded history—we have learned much about pandemic influenza and the origins of antigenic drift among influenza A viruses. Despite this knowledge, we remain largely underprepared for when the next major pandemic occurs.

While emergency departments are likely to care for the first cases of pandemic influenza, intensive care units (ICUs) will certainly see the sickest and will likely have the most complex issues regarding resource allocation. Intensivists must therefore be prepared for the next pandemic influenza virus. Preparation requires multiple steps, including careful surveillance for new pandemics, a scalable response system to respond to surge capacity, vaccine production mechanisms, coordinated communication strategies, and stream-lined research plans for timely initiation during a pandemic. Conservative models of a large-scale influenza pandemic predict more than 170% utilization of ICU-level resources. When faced with pandemic influenza, ICUs must have a strategy for resource allocation as strain increases on the system.

There are several current threats, including avian influenza A(H5N1) and A(H7N9) viruses. As humans continue to live in closer proximity to each other, travel more extensively, and interact with greater numbers of birds and livestock, the risk of emergence of the next pandemic influenza virus mounts. Now is the time to prepare and coordinate local, national, and global efforts.

## Background

In this literature review, we aim to summarize current knowledge of preparation and potential management for a pandemic influenza virus. With increasing travel, immigration, crowding, and human interaction with livestock, there is an ever-increasing risk of another pandemic. We specifically focus on how intensive care units (ICUs) and their staff may prepare for such an event.

Seasonal influenza has had a long history with humans, but at several points in history, a novel strain of influenza will emerge and lead to a pandemic. A pandemic is an epidemic of disease that has spread across a large region, or even worldwide. There have been four influenza pandemics in the past century, and the circumstances of their emergence are described in this paper.

We outline major steps to prepare for a pandemic including (1) surveillance for new pandemics, (2) building a scalable system to respond to surge, (3) the mass production of vaccines, (4) integrated and coordinated communication, and (5) harmonized research and ethics proposals for rapid initiation. A serious influenza pandemic is very likely to overwhelm the health care system. We describe triage strategies and approaches when resources are limited.

## History and pathogenesis of pandemic influenza

**Fig. 1:**
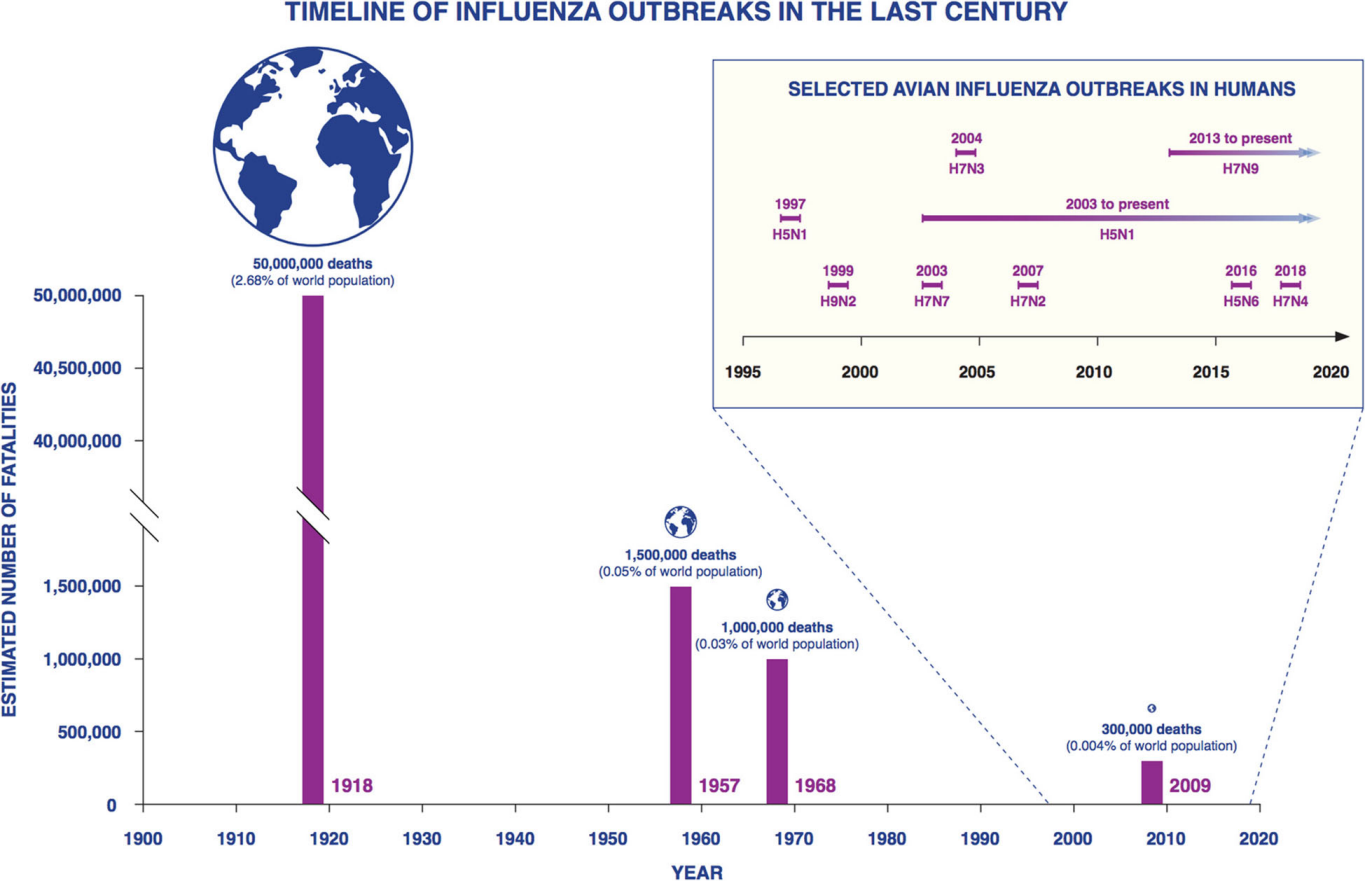
Timeline of recorded influenza outbreaks in the past century as well as selected avian influenza outbreaks in humans. In the main figure, globe size corresponds to the estimated number of fatalities relative to global population size at the time, with vertical bars representing the estimated absolute number of fatalities. Numbers are averages from various approximations. In inset, horizontal bars represent time frame that cases from the influenza A virus strain were recorded

There may be no virus that has shaped human history and mortality more than influenza. We now mark the hundredth anniversary of the deadliest influenza outbreak recorded—the 1918–1919 “Spanish influenza”—which claimed an estimated 50 million lives [1]. Since the Spanish influenza, pandemics have become an increasing threat with more frequent movement of people and pathogens (Fig. 1).

Pandemic influenza arises distinctly from seasonal influenza. Seasonal viruses circulate globally and evolve due to point mutations in the genetic sequence resulting in small changes in two surface glycoproteins—hemagglutinin (H) and neuraminidase (N). Both influenza A and B undergo this process, known as antigenic *drift*, leading to a recommendation for yearly influenza vaccination [2]. Due to its segmented genome, influenza A also has the unique ability to undergo more significant rearrangements, known as antigenic *shifts*. Antigenic shifts are necessary, but not sufficient, for pandemic influenza to occur, and they usually result in new circulating strains of seasonal influenza viruses. Only influenza A virus is known to have caused pandemics. Influenza C can be a cause of acute respiratory disease in children, but rarely in adults [3]. Studying the development of the four major influenza pandemics of the last hundred years—in 1918–1919, 1957–1958, 1968–1969, and 2009–2010—provides insights into how pandemic influenza may next occur.

The origin of the 1918 “Spanish influenza” remains controversial. Before the genome was decoded by Taubenberger et al. [4], the virus was considered to be derived directly from avian origin [5, 6]. With available genetic information, phylogenetic analysis showed the 1918 pandemic strain contained more similarities with mammalian lineages, either swine or seasonal human H1N1 virus. While debate exists, Smith et al. further showed the 1918 strain resulted from reassortment of genes of circulating swine and human influenza viruses with introduced avian viruses over several years, rather than direct adaptation of an entire avian virus [7]. Regardless of exact etiology, the 1918 pandemic influenza caused devastation in a world still struggling from the Great War. Crowding—of soldiers and civilians—affected the spread of influenza and severity of the illness [ 8, 9]. These crowded conditions provided ideal conditions for a novel influenza strain to become a pandemic, which spread globally as soldiers returned home at the end of the war [10].

It was four more decades before the world faced another two influenza pandemics in short succession. The 1957 and 1968 pandemic viruses formed from genetic reassortment. The 1957 “Asian influenza” H2N2 virus resulted from reassortment between low-pathogenic avian influenza (LPAI) H2N2 and seasonal H1N1 virus, while the 1968 “Hong Kong influenza” H3N2 virus resulted from rearrangement of LPAI H3N2 and the seasonal H2N2 virus circulating since the 1957 pandemic [11, 12]. The 1957 “Asian influenza” pandemic caused an estimated 1.1 million excess deaths due to respiratory disease—two thirds in individuals under 65 years old [13]. The 1968 pandemic killed an estimated 1 million individuals [14]. These estimates also under account for mortality in resource poor settings which have less capacity for microbiological testing and documentation.

In March and April 2009, the first pandemic influenza virus of the twenty-first century began to circulate in Mexico and the USA. H1N1pdm09 virus was a novel influenza virus strain in humans. The virus was a combination of Eurasian and North American swine lineages. The majority of the genes were derived from H3N2 and H1N2 triple reassortment viruses in pigs, while their neuraminidase genes were derived from a wholly avian influenza virus that entered the Eurasian swine population [15]. The resulting “swine flu” was distinct from circulating seasonal influenza A viruses, and younger individuals had little or no natural immunity. Mortality globally was estimated between 151,700 and 575,400 in the first year of circulation. Eighty percent of H1N1pdm09-related deaths were in individuals under 65 years, compared to 10–30% in seasonal influenza outbreaks [16].

We have seen pandemic influenza occur multiple times before, and at increasing rates. History has shown us how devastating pandemic influenza can be, especially to younger, healthier individuals. There appears to be an increasing number of pandemics, which is only likely to worsen with growing human population, crowding, and immigration. When considering preparation for the next pandemic, it is not a matter of if it will occur, but rather a matter of when.

## Preparation for a pandemic

Despite attempts at planning, we remain unprepared. Following the 2009 pandemic, the International Health Regulations committee concluded that “the world is ill-prepared to respond to a severe influenza pandemic or to any similarly global, sustained, and threatening public-health emergency” [17]. If we are unprepared to deal with pandemic influenza in developed nations, this pales in comparison with developing nations. By almost all accounts, “Sub-Saharan African plans are not ready to prevent or reduce the death count from [pandemic] influenza” [18, 19]. Intensive care unit (ICU) mortality during the 2009 pandemic varied substantially not only with patient characteristics but also based on region and economic status of the outbreak location; the highest mortality experienced was in South Asia and sub-Saharan Africa [20]. If we are to better prepare for pandemic influenza, it will require multiple components:

1. *Careful surveillance to recognize and mitigate new pandemics*—Controlling pandemics requires early recognition to curb the spread of novel viruses; this necessitates a coordinated surveillance and reporting system. Following the 2009 pandemic, the WHO attempted to mitigate shortcomings by adopting the Pandemic Influenza Preparedness Framework [21], which created sentinel sites for seasonal influenza and to monitor for unusual events that may herald novel influenza. While most surveillance occurs outside of ICUs, with non-critically ill patients, intensivists can still perform a vital function in surveillance of severe disease. In 2009, we saw that our previous reporting systems were not dependable; they relied on patients presenting to physicians, which is influenced by public alarm among other factors. Initial case fatality rates for H1N1 differed by up to 50-fold [22]. Conversely, ICU admission criteria are relatively fixed over time. Cases and deaths can be easily tracked, making ICUs ideal places for surveillance of severe pandemic influenza. To use this strategy, it will be important that intensivists understand the size of their catchment (or referral) area so that they can accurately estimate the local incidence. The creation of early warning systems was one of the main goals of the International Forum for Acute Care Trialists (InFACT) and ongoing efforts such as the SPRINT SARI study [23].
2. *An efficient and scalable emergency response system that can respond to surge capacity*—Pandemic preparedness relies on a system that can surge in times of crisis. Surge capacity has four key components: equipment, physical space, human resources, and system [24]. In pandemics, the duration, scope, and magnitude of the response required are uncertain. In most countries, health care systems operate at or above maximally designed capacity. Many hospitals just do not have sufficient pre-existing resources to respond to surge capacity in an outbreak [25]. Unlike with natural disasters, where the greatest need for resources often occurs early in the time course, pandemic resource requirements will build over months. Outbreaks that become pandemics generally do not take hold in multiple locations at exactly the same time—they are geographically and temporally patchy. Still enough must be immediately available to allow time for other regions and/or manufacturers to meet the increased demand.

Estimates of capacity required in a severe pandemic vary widely. Using the “Flu Surge” model [26] and assuming 35% attack rate over 6 weeks, in Canada’s most populous province, Ontario, it is predicted that influenza patient admissions would peak at 1823 per day, which is 72% of all hospital capacity just for influenza patients alone. Demand for ICU resources would peak at 171% of current ICU bed capacity, and ventilator use would peak at 118% capacity. These numbers would only add to the region’s current day-to-day ICU utilization rates, which are approximately 90% capacity [27]. In Canada, this would definitely overwhelm current ICU resources. During the 2009 pandemic, in Canada, there were only 3170 ICU beds and 4982 ventilators—a median of 10 ICU beds capable of providing invasive ventilation and 15 ventilators per 100,000 persons [28]. Therapies to treat the most severely affected patients were available in a minority of centers—inhaled nitric oxide in 79 (27.6%) and extracorporeal membrane oxygenation (ECMO) in 39 (13.6%). The uncertainty in scope however leads to uncertain estimates. Models often provide no more accurate estimate of need than expert consensus [29]. In a systematic review of disaster surge capacity, most studies classified an increase in surge capacity of 15–35% as “acceptable,” [25] likely far short of what would be required, and certainly short of the *CHEST* consensus statement recommendations of 200% [30]. These estimates also do not account for loss of capacity due to health care worker illness, which we know from previous pandemics and outbreaks can be significant [31].

Even in most well-developed countries, ICU beds are often close to capacity, and it is likely that in a severe influenza pandemic many patients who require a ventilator may not have access to one. Severe acute respiratory distress syndrome (SARS) gave a small-scale example of this. SARS resulted in 8096 cases globally, with only 251 in Canada [32]. Despite this, resources were critically stretched. In Ontario, every negative pressure room in the province was occupied with more patients awaiting at home during the height of the pandemic [33]. ICUs should expand into other areas in a tiered method to facilitate increased demand, with appropriate training of new staff occurring rapidly during times of surge. Intensivists must advocate, and lead, a proactive response with our health care bodies in planning and budgeting for potential surges.

3. *The ability to efficiently and quickly mass produce and distribute vaccines—*Vaccination readiness remains a mainstay of preparation for pandemic influenza, but relies mainly on the efforts of influenza researchers and public health authorities. Details of this are discussed in other reviews [34–36]; briefly, once pandemic influenza is recognized, production of a vaccine will begin. Meanwhile, a priming dose can be considered if stockpiled in specific countries. Once candidate pandemic vaccines are produced, observational studies and clinical trials for safety and efficacy should ideally occur before or alongside their introduction to the clinical setting. This process is inherently long, and measures to streamline the process are vital.
4. *Integrated and coordinated communication—*Excellent communication is vital to a timely response to a disaster scenario. Hospitals and hospital networks should appoint local leads and teams that will respond and coordinate during a pandemic. There should also be secure online directories of all key partners’ contact information and clinical and administrative positions. Teams should meet regularly to sharpen communication and build trust, with annual inter-outbreak meetings being the minimum recommended to develop effective relationships [30]. We have seen on a much smaller scale this work with local trauma networks. Hospitals regularly run disaster scenarios, yet these rarely extend beyond the first few hours of an emergency. Broader scenario training or simulation of pandemics is vital to preparedness.
5. *Coordinated research plans with pre-approved research ethics to allow timely initiation—*A well-structured research program is paramount to learn and adapt as pandemic influenza develops. Research during a pandemic must be partially predetermined, have accelerated research ethics vetting, and be pragmatic. Recent pandemics have been characterized by an inability to efficiently undertake interventional trials necessary to guide best practices [37]. The first clinical research step during a pandemic will be descriptive using pre-existing case report forms and formulating an accepted case definition [38]. Most large jurisdictions already have pre-approved tiered case report forms, with minimal or expanded versions, so they may serve as data collection tools for clinical trials [39]. Funding agencies must also provide shortened intervals from application to approval, ideally with prepositioned funds for immediate vetting and release. Finally, there should be coordinated communication of research interests and intent across global regions at the outset to promote complementary and generalizable results without unnecessary duplication in efforts [40].

## Intensive care and hospital management during a pandemic

While emergency departments are likely to encounter the first patients with pandemic influenza, many sick patients should be cared for by intensivists, so they are critical to guiding triage when demand exceeds capacity. Intensivists therefore should be part of strategic planning committees before, during, and after pandemics, to coordinate ICU response with hospital and regional efforts for triage, clinical care, and infection control.

**Fig. 2:**
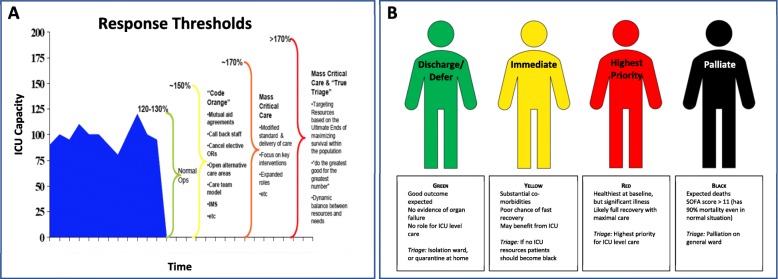
**a** Stages of mass critical care, with various ICU response thresholds. As a pandemic progresses, resources become scarce and there is increasing strain placed on the health care system from more cases [24]. **b** A potential triage strategy for various patient groups as the capacity of the ICU is slowly overwhelmed to streamline admissions without the greatest opportunity for benefit from ICU level care. Transparency is paramount in this process

During a large-scale pandemic, resources will become limited, even in developed nations. Multiple and context-appropriate strategies will be required to build a sustained surge capacity for mass critical care. While short-term capacity is crucial, long-term sustainability will be more important. The starting point for this in Canada is the Canadian Pandemic Influenza Plan [41]. In the USA, these include, among others, Pandemic Influenza: Preparedness, Response, and Recovery from the Department of Homeland Security [42], and the Pandemic Influenza Plan from the CDC and Department of Health and Human Services [43]. Clinicians must be adaptable when using pre-existing protocols, as they are often based on historical and non-generalizable illness syndromes and outcomes. Resource-limited countries will also need significant adaptation, likely with a greater focus on pre-hospital and transportation systems [44] (Fig. 2).

Treatment of severe influenza involves a combination of specific and supportive therapies. While there is limited evidence of the effectiveness of neuraminidase inhibitors in severe influenza, they are likely to be recommended for use in critically ill patients during the initial phases of pandemic influenza [41–43]. Pandemic influenza should also be treated according to the pathophysiological mechanism of injury. While influenza results mainly in upper and lower respiratory tract infection, secondary bacterial pneumonias, acute respiratory distress syndrome (ARDS), encephalitis, and myocarditis complicate severe illness. Many patients will require mechanical ventilation. If demand outstrips critical care capacity, a triage system will be needed in developed health systems; this already routinely occurs in resource-limited settings. Developing a pandemic-specific and responsive triage system has proven challenging even in highly resourced systems. Triage systems based upon the severity of illness scores, beyond which intensive care might be considered futile, are fraught with poor performance for individual patient decisions and were not developed involving the patients to whom the triage tool would be applied. For example, the 2009 pandemic affected young non-immune patients, many of whom had high illness severity scores; however, with intensive care, mortality was low in developed countries [45]. Modeling data suggests that to perform better than a first-come, first-served basis, the triage tool would have to have a 90% sensitivity and specificity [46]. The Ontario Health Plan for an Influenza Pandemic critical care triage protocol assembled a task force with public consultation to determine the best distribution of resources during a pandemic. Surprisingly, only “first-come, first-serve” and “random selection” principles were favored by the panel, based on a need to balance a utilitarian approach with equity considerations. They suggested that “these criteria serve as a defensible ‘fail safe’ mechanism for any triage protocol” [45] (Table 1).

**Table 1:** Outline of possible triage strategies during a pandemic or other emergency situation where resources are limited. Multiple task forces favor FCFS and traditional methods as the most ethical during a pandemic

Method	Mechanism of medical triage	Prioritizing factor	Examples
Traditional	No formal mechanism of triage	No criteria	Many health care systems
Barron Dominque-Jean Larry	Treatment of the most urgent (i.e., sickest) patients, and deferring less sick or likely fatal cases	Market pull factor	How current system works in most of the developed world
Wilson	Concentrate treatment on the most likely to be successful. Some low probability cases will die that otherwise may have been saved	Likelihood of success	Pragmatic approach
First-come, first-served (FCFS)	Treatment based on arrival/presentation regardless of severity of illness, rank, or any other criteria	Order of arrival	In part, how current system works in most of the world
Greatest good for greatest number (GGGN)	Depriving severely ill patients needing large amount of resources and attention, for multiple patients that are less sick and require less resources	Number of patients treated for given resources	Utilitarian approach
Less severity first treatment (LSFT)	Prioritize healthier patients that can be treated quickly to allow them to return to society, the labor force, etc.	Patients who are less sick	Many emergency departments have a fast track section
Maximize the fighting strength	Treat patients who are most likely to quickly return to duty with the least resource expenditure	Time needed for treatment of patients	Prioritize HCWs, key public health or government jobs, etc.

Beyond mechanical ventilation, access to extracorporeal life support (e.g., ECMO) will be an even more limited, but perhaps life-saving, resource during a pandemic [47]. There may be barriers to patient transfer between institutions given infection control concerns, limiting access to treatment. Mobile units capable of setting up ECMO at peripheral sites before transfer may be preferable during a pandemic and was a successful approach used during the 2009 H1N1 pandemic [48]. While ECMO appears to be effective in the treatment of selected patients with severe ARDS [49–51], it relies on a smaller scale pandemic. In the event of a pandemic that overwhelmed the health care system, existing ECMO resources might be allocated using existing locally acceptable criteria, coupled with a first-come, first-served basis, understanding that in a sustained outbreak, time-limited trials of treatment represent one mechanism to effect triage.

During a severe pandemic, context-appropriate standards of care would be required if demand for resources substantially exceeds capacity. Such a crisis-based standard of care might be defined as a “substantial change in usual healthcare operations and the level of care it is possible to deliver, which is made necessary by a pervasive … or catastrophic disaster” [52]. The release of crisis standards of care would be made by the regional or national governments, through Ministries of Health or Public Health Agencies, but intensivists would reasonably be expected to be involved in this process of development. Such standards might consider (1) mechanical ventilation, (2) IV fluid resuscitation, (3) vasopressor administration, (4) sedation and analgesia, (5) antiviral treatment, and (6) therapeutics and interventions, such as renal replacement and nutrition for critically ill patients [29]. Thought should also be placed on dealing with special populations—such as children and pregnant women [30].

While providing high levels of critical care through a pandemic, we must maintain the safety and wellbeing of health care workers (HCWs). Beyond any professional obligation to HCW safety, there is also likely to be a public health benefit to this—when HCWs become sick, or fear becoming sick, they are less able to perform clinical duties. Lessons can be learned from experiences in Toronto and other major centers with SARS. Approximately 20% of cases globally were in HCWs [53]. Nosocomial amplification is a common aspect of many outbreaks. While influenza is regularly spread through contact and droplet transmission, certain procedures in hospitals—intubation, ventilation, and bronchoscopy—create potential airborne transmission. Infection control practices are essential to limiting the spread of pandemic influenza [54]. The loss of clinical personnel to illness resulted in the shutdown of most non-urgent healthcare for the entire city. Preventing this loss of capacity by protecting health care personnel is a critical element of an effective response.

Public health officials working with clinical experts must make rapid recommendations about appropriate personal protective equipment, and for novel threats, these recommendations must be updated as more information about the pandemic becomes available. Pre-pandemic simulations can play a vital role in preparing staff for these outbreaks—for infection prevention and control, for clinical care practices, and also to help staff prepare “emotionally” for stressful environments.

We can also design ICUs to limit the spread of infection. In Singapore, following SARS, the emergency room was redesigned so that febrile patients were allocated where air flow patterns did not carry to other areas of the department [55]. In Toronto after SARS, the intensive care unit at the main outbreak center was rebuilt with an entire pod of beds that could be converted into a negative pressure ward. These designs and many others will help manage the next outbreak and these factors should be considered when all new hospitals are being constructed. During a pandemic, visitors and non-essential personnel should likely be limited in hospital entry, while respecting the needs of patients and families to safely connect—either in person with appropriately supported PPE or using novel ward design and/or electronically augmented virtual connections.

## Our current landscape

The US Department of Homeland Security “views pandemic influenza as both the most likely and the most lethal of all [infectious] threats facing the United States,” [56] a concern shared by many health jurisdictions [57]. Interpandemic periods average 40 years, but we are at an ever-increasing risk for serious pandemics [58]. As humans continue to live in more crowded conditions, travel and migrate more extensively, and continue to farm livestock in proximity to more densely populated areas, the risk for genetic reassortment of influenza A viruses is perhaps higher than ever before.

As outlined above, the most recent pandemic influenza virus, in 2009, originated from pigs. While swine will remain a major concern for further pandemics, birds likely pose the greatest risk for deadly pandemic influenza virus strains. Like pigs, they serve as reservoirs and can be infected with multiple strains making them a potential mixing vessel [59]. There are several strains of high-pathogenic avian influenza (HPAI) that pose the greatest threat to humans [60]. In 1997, Hong Kong reported the first outbreak of influenza A(H5N1) in humans. Virus was transmitted from chickens directly to humans, and 6 of 18 patients died [61]. Since 2003, the virus strain has spread to Europe and Africa killing millions of poultry and causing hundreds of human infections. While there has been no sustained human-to-human transmission of H5N1, the overall mortality rate is close to 60%. In 2013, a novel avian influenza A virus, H7N9, emerged and began to spread across poultry in China. H7N9 has resulted in over 1500 human cases with a 40% mortality rate [58]. Most of those infected in recent outbreaks could reasonably be expected to receive care in an ICU.

Global hot spots for emerging infectious diseases and pandemic influenza are often in some of the regions with the least resources. Many countries where HPAI remains a major pandemic threat have limited participation (data generation, genetic analysis, data share, etc.) in avian influenza surveillance [62]. In addition, some countries may have political, economic, or scientific disincentives to share surveillance data gathered [63].

The 2009 H1N1 pandemic was, by most accounts, not as severe as initially feared. Many have therefore become complacent about the prospect of an influenza pandemic. However, it should be noted that 5 months after the discovery of the novel virus in Mexico, 50% of children in Hong Kong were infected with H1N1, proving rapid dissemination of a pandemic virus [64]. Vaccines cannot be developed in time to protect against the first wave of a novel pandemic and should a deadlier virus, such as HPAI, spread at this rate, the results would reflect those seen in a Hollywood movie. We are unprepared at a local level in ICUs and at a global public health level for such a situation. Now is the time to act in our own hospitals and to use our influence to help guide government policies.

## Conclusions

The threat of a new influenza pandemic remains high. Health care systems, and intensive care units, around the world are at risk of clinical demand outstripping capacity. Action should be taken now to build surveillance systems, a scalable response with focus on vaccine production, effective cross-jurisdictional communication and clinical support, the potential to require fair and effective patient triage systems, in addition to research embedded within a pandemic plan.

## Data Availability

Not applicable.

## Abbreviations

H: Hemagglutinin
N: Neuraminidase
LPAI: Low-pathogenic avian influenza
HPAI: High-pathogenic avian influenza
ICU: Intensive care unit
SARI: Severe acute respiratory infection
ECMO: Extracorporeal membrane oxygenation
CDC: Centres for Disease Control
ARDS: Acute respiratory distress syndrome
FCFS: First-come, first-served
GGGN: Greatest good for the greatest number
LSFT: Less severe, first treatment
HCW: Health care workers
